# Their voices matter: assessing patients’ experience with healthcare quality in a war-torn country

**DOI:** 10.1186/s13031-025-00739-0

**Published:** 2025-12-08

**Authors:** Swsan A. M. Elsharif, Anfal M. Altahir, Asma Y. Mohammed, Nawran Zakaria, Anfal Khalid Abdalla Mohammed, Afaq Shannan, Omar Abdelhaleem, Ragda Bashir, Suad Sh. Hassan, Suodad Elhassan, Elaf M. H. Abdelraheem, Abu-Bekr Mohamed, Marwa Osman

**Affiliations:** 1 Faculty of Medicine, University of Khartoum, Khartoum, Sudan; 2 Faculty of Medicine, University of Ahfad, Omdurman, Sudan; 3 Faculty of Medicine, University of Alzaeim, Alazhari, Sudan; 4 Faculty of Medicine, University of Bakhat Alruda, Ad Douiem, Sudan; 5 Faculty of Medicine, Department of Community Medicine, University of Khartoum, Khartoum, Sudan

**Keywords:** Quality of care, Patient experience, Displacement, War, Humanitarian settings, Quality in crisis, Health system.

## Abstract

**Background:**

Sudan is experiencing the largest displacement crisis globally due to the ongoing war. One key displacement area is the White Nile state. Despite the state’s relative safety, the indirect effects of the war are felt in its health system. In conflict and fragile settings, patients’ experiences with healthcare quality receive little or no attention. Thus, our study aimed to assess the experience of patients admitted to governmental hospitals in White Nile state.

**Methods:**

The study took place in the largest cities in the White Nile State: Kosti, Rabak, and Ad Douiem. A total of 799 patients from Medicine, Surgery, Obstetrics and Gynaecology wards were interviewed from September to October 2023 using the In-Patient Assessment of Health Care survey, which addresses communication with doctors and nurses, the physical environment, medication communication, pain management, and the overall care.

**Results:**

A total of 799 participants were interviewed. The median age was 28, and most (85.2%) were females. The internally displaced persons (IDPs) were (20.7%), and most of them were staying in host communities (69.1%). The overall quality of care rating was relatively low, with only (19.5%) rating the hospital as “5” best. About (82.4%) of the patients would recommend the hospital. Regarding the predictor of the overall rating of quality, physical environment was found to be a strong predictor (OR 1.337; 95% CI 1.208–1.480; *p* < 0.001). Regarding recommendations of the hospital, IDPs were found to have a lower tendency to recommend the hospital (OR 0.561; 95% CI 0.358–0.881; *p* 0.012).

**Conclusion:**

The overall rating of quality of care was relatively low. However, most patients would recommend the hospital. This contradiction may highlight their low expectations of the health system. Such a perspective must drive the efforts of healthcare providers and policymakers to improve patients’ experiences and thus improve patient-centred care.

**Supplementary Information:**

The online version contains supplementary material available at 10.1186/s13031-025-00739-0.

## Introduction

Quality of care is defined as “the degree to which health services for individuals and populations increase the likelihood of desired health outcomes”. It is also defined as care that is safe, effective, timely, efficient, equitable, and patient-centred. Patient-centred care is defined as “care that is respectful of and responsive to individual patient preferences, needs, and values, and ensuring that patient values guide all clinical decisions [1]. Patient-centred care has many measures, one of which is patient experience, which is a process measure [2, 3].

The Beryl Institute defines patient experience as “the sum of all interactions, shaped by an organisation’s culture, that influence patient perceptions, across the continuum of care [4]. Patient experience examines both the effectiveness of healthcare and the humanity of care, such as dignity, respect, privacy, communication, emotional support, cleanliness of the facilities, delays and waiting times, and can outline many areas for improvement [5–7]. Patient experience and satisfaction (the degree between expectation and experience) with healthcare services were confirmed to influence their clinical outcomes and quality of life [8].

Quality of health care should be provided in all settings, as “leaving no one behind” became the core technique to achieve sustainable development goals and universal health coverage [2, 9]. Thus, it should be assessed in fragile and conflict-affected situations, ensuring that all individuals receive high-quality care regardless of their background [2, 10]. Quality of care in humanitarian settings needs more focus. A systematic review of the quality of care during crises suggested that there is a failure to provide patient-centred care in humanitarian settings. It also highlighted the need for the adaptation of new quality improvement measures that focus on system competence, patient experience and health outcomes [10].

Sudan’s health system possesses a lot of strength points in terms of an adequate number of health workers, policy and basic package of health services availability; however, it has been suffering from the poor logistic supply, poor data quality, referral system, and managerial system for coordination between stakeholders, in addition to low financial spending on health with the long-standing economic instability and outdated health legislations [11]. This system also features an inequitable healthcare distribution of the workforce, as about 70% of them are working in urban settings, serving only about 30% of the Sudan population [12]. Inequities and challenges of healthcare provision are typically expected to expand after the beginning of Sudan war in 2023; the Federal Ministry of Health (FMOH) has previously identified the internal conflicts in Sudan as one of the primary causes of the deterioration of healthcare [13]. The ongoing war in Sudan has not only resulted in direct disruption to healthcare service delivery in conflict sites through attacks and indirect compromise, but also the large displacement trend -internally- has left the previously underfunded health system overwhelmed. This high rate of displacement remains disproportionately higher than the capacity of the health system in regions receiving the displacement flow [14, 15]. This war compromised the health system, including country regions with no active armed conflicts or direct attacks on health infrastructure, as this war has exacerbated the burden of communicable diseases, in addition to malnutrition and mental illness, as well as disrupted workforce deployment and distribution. Moreover, further challenges are expected as a further shrink is anticipated due to the economic stress and shifted investment in the military domain [15].

Sudan’s health system has nearly collapsed due to the ongoing armed conflict. Although White Nile state was not directly affected by the conflict, it is suffering from the continuing surge of refugees and internally displaced persons, along with the reports of disease outbreaks, malnutrition, and the continuing shortage of medical supplies due to transportation and financial constraints [16, 17]. Through this time of critical change, it is necessary to reassess outcomes and outputs after the forced acute changes and consequent patient experience with the health system and care provision in White Nile state. This can efficiently redirect policy-making efforts toward more patient-centred care that is more likely to meet patients’ needs.

## Methods

### Study design

This is a descriptive, cross-sectional, facility-based study, conducted from September 10th to October 24th, 2023. Study reporting followed the STrengthening the Reporting of OBservational studies in Epidemiology (STROBE) guidelines [18].

### Setting and participants

The study was conducted at the three main hospitals in White Nile State, namely Kosti, Rabak, and Ad Douiem. White Nile State is located on the border between Sudan and South Sudan and covers around 150,000 square kilometres. With South Sudan to the south and Khartoum to the northeast, the state is a major shelter for refugees and displaced people.

The hospitals were chosen deliberately because they cover a large proportion of the population in the three largest cities in White Nile State and the surrounding region.

#### Kosti teaching hospital

Kosti Teaching Hospital is the main hospital in the state, serving a large population with a high admission rate of 2000 patients per month. The hospital has a capacity of 196 beds. It provides a wide range of services, including intensive care, oncological consultation, and chemotherapy, as well as psychiatric services.

#### Rabak teaching hospital

Rabak Teaching Hospital is the biggest hospital in Rabak locality, with an average monthly admission rate of 617 patients. The hospital has a capacity of approximately 93 beds.

#### Ad Douiem teaching hospital

Ad Douiem Teaching Hospital is the main hospital in Ad Douiem locality, with an average admission rate of 483. The hospital has approximately 60 beds.

### Participants

The study population comprised all inpatients admitted to any of the three hospitals.

**Inclusion criteria**:

- Participants must be 18 years old or older.
- Participants must be mentally and physically capable of providing ethical consent.

**Exclusion criteria**:

- Patients who were admitted to the private section of the hospital.
- Patients who were admitted to the short stay for less than 24 h.
- Patients who were admitted to the ICUs were not included due to the infection control and patient safety measures.

### Sample size

To calculate the sample size, we used Yamane’s simplified formula for a single population proportion N/(1 + Ne2), with a margin of error (e) of 0.05. We considered each hospital as a stratum and obtained the average number of inpatients admitted to the Surgery, Medicine, and Obstetrics and Gynaecology (OB/GYN) wards over six months. The average number of admissions (N) was used to calculate the required sample size of each hospital using the formula. The sample size for each hospital was then proportionally allocated to the different wards based on their share of admissions (see supplementary materials). Then the total sample size was summed up to obtain 722 participants. Then we add 10% for potential non-response and to account for incomplete response, which gave 794. We included 799 eventually, as increasing the sample size in non-probability sampling increased the power of the study [19, 20].

### Sampling technique

We employed a consecutive sampling technique to collect data from the hospitals. Consecutive sampling involves obtaining samples on a first-come, first-served basis after confirming that they meet all inclusion criteria. The recruitment process continues until the desired sample size is achieved. This technique was chosen due to the difficulty in applying systematic random sampling, as the hospitals lack clear and updated patient databases, well-documented admission processes, patient ID numbers, and numbered beds.

### Study variables

#### Independent variables

Age, gender, marital status, residency, education, occupation, monthly income, means of transportation, distance from residency to hospital, admission type, reasons for admission, payment status, and health insurance, in addition to I-PACH survey items.

#### Dependent variables

Overall rating of quality of care, as well as the recommendation of the hospital.

## Data collection method

### Instrument

The primary instrument used for data collection was the Patient Assessment of Healthcare for Inpatient Care (I-PAHC), originally developed and validated in Ethiopia [21]. This instrument is specifically designed for use in low-income countries and was considered the most suitable for application in Sudan. The I-PAHC covers five domains of care: nurse communication, doctor communication, physical environment, pain management, and medication communication. Responses were recorded on a 4-point Likert scale, ranging from 1 (never) to 4 (always). Additionally, socio-demographic questions were included.

The questionnaire underwent a strict revision process by the supervisor, a social worker, and experts in quality of care. It was then translated forward translation to Arabic by the author and then translated to the local accent; backwards translation to English by a native speaker; and a pilot study was conducted with 12 patients, after which the questionnaire was revised based on patient feedback and re-evaluated by field experts.

### Techniques

Seven data collectors received comprehensive training on research methodology, including proposal writing, literature review, and data collection techniques. They were also briefed on the study’s purpose and trained in conducting face-to-face interviews. The KoboToolbox application, an offline mobile data collection app for epidemiological surveys, was explained and utilised for data collection. Data was gathered through face-to-face interviews and recorded in the KoboToolbox.

### Data analysis

The data was transferred from Kobo Collect to an Excel spreadsheet, then manually cleaned variable by variable, coded, and input into SPSS version 20 for analysis. Descriptive statistics were used to describe patient characteristics and experiences. To test the relationship between the sociodemographic and hospitalisation characteristics with the first dependent variable, which is the overall rating of care, Kruskal-Wallis test and Mann-Whitney test were employed, and to test the relationship between patient experience factors (continuous) and overall rating of care, Spearman’s correlation test was used. For the association between independent variables and the second dependent variable, which is the recommendation of the hospital, the chi-square test was used. P-values < 0.05 were considered statistically significant.

Binary logistic regression models were used to further identify the predictors of the dependent variables, plotting only the significant variables. The first dependent variable (overall rating of care) was dichotomised to 1 (4,5 best) and 0 (1 worst, 2, 3) for analysis purposes. The second dependent variable (recommendations) was coded to yes: 1, and no: 0.

### Ethical considerations

Ethical approval was obtained from the White Nile State Ministry of Health Review Board (approval number 023081) and the Department of Community Medicine, University of Khartoum (approval number COMMED 2023-95-20). Administrative approval was secured from each hospital. Informed consent was obtained from all participants, who were clearly informed of the research purpose and their right to withdraw from the study at any time. Data were anonymised and handled confidentially by the research authors.

## Results

### Sociodemographic characteristics

A total of 799 study participants were included in the study. The median age was 28 years, and the majority (85.2%) were females. Most (92.7%) were married. A significant portion of participants (57.1%) received primary education or lower, and only (12.6%) of them had higher degrees. Regarding occupation, (80.2%) of participants were non-employed, and (71.3%) of them had an income level of 100,000 SDG and below. In addition, (49.2%) of them came from rural settings and (51.7%) travelled more than ten kilometres to access the hospital. Most (61.1%) came by public transportation, and (50.9%) spent one hour or more to reach the hospital. Most importantly, (20.7%) of the study participants were internally displaced from conflict-affected areas (Table 1). Most of them were displaced from Khartoum (90.9%) and were staying in host communities (69.1%). (Appendix Table 1).

Kruskal-Wallis and Mann-Whitney U tests were used to examine the association between the overall rating of quality of care and socioeconomic characteristics. None of these characteristics showed a significant association with the overall quality of care (*p* > 0.05).

In contrast, Chi-square test was used to assess the relationship between the recommendation of the hospital and sociodemographic characteristics. Gender, educational status, residence, distance to the hospital, time taken to reach the hospital, transportation, and displacement status were all found to be significantly associated with recommendation of the hospital (*p* < 0.05) (Table 1).

**Table 1:** Sociodemographic characteristics of the study participants admitted to Kosti, ad Douiem, Rabak teaching hospital, white nile state, 2023, N: 799

Characteristics	Overall *N*: 799		Recommendation					The overall rating of quality of care	
			No		Yes		*P* value^a^	Median (IQR)	*P* value
**Age**	28 (23–35) *						0.338		0.991^b^
18–34	582	(72.8%)	96	(16.5%)	486	(83.5%)		4 (3–4)	
35–64	202	(25.3%)	42	(20.8%)	160	(79.2%)		4 (3–4)	
≥ 65	15	(1.90%)	3	(20.0%)	12	(80.0%)		4 (3–4)	
**Gender**							**0.045**		0.663^c^
Male	118	(14.8%)	29	(24.6%)	89	(75.4%)		4 (3–4)	
Female	681	(85.2%)	112	(16.4%)	569	(83.6%)		4 (3–4)	
**Marital Status**							0.060		0.555^c^
Married	741	(92.7%)	125	(16.9%)	616	(83.1%)		4 (3–4)	
Unmarried	58	(7.30%)	16	(27.6%)	42	(72.4%)		4 (3–4)	
**Educational Status**							**0.004**		0.059^b^
Lower than primary	179	(22.4%)	24	(13.4%)	155	(86.6%)		4 (3–4)	
Primary School	227	(34.7%)	39	(14.1%)	238	(85.9%)		3 (3–4)	
Secondary School	242	(30.3%)	50	(20.7%)	192	(79.3%)		4 (3–4)	
University and above	101	(12.6%)	28	(27.7%)	73	(72.3%)		3 (3–4)	
**Occupational status**							0.050		0.688^b^
Non employed	641	(80.2%)	103	(16.1%)	538	(83.9%)		4 (3–4)	
Employee (Governmental/Private)	41	(5.10%)	13	(31.7%)	28	(68.3%)		3 (3–4)	
Farmer or daily labourer	95	(11.9%)	20	(21.1%)	75	(78.9%)		4 (3–4)	
Student	22	(2.80%)	5	(22.7%)	17	(77.3%)		4 (3–4)	
**Income in SDG/month**							0.240		0.204^b^
< 50,000	318	(39.8%)	46	(14.5%)	272	(85.5%)		3 (3–5)	
50,000–100,000	252	(31.5%)	51	(20.2%)	201	(79.8%)		3 (3–4)	
100,000–150,000	141	(17.6%)	25	(17.7%)	116	(82.3%)		4 (3–4)	
150,000–200,000	46	(5.80%)	8	(17.4%)	38	(82.6%)		4 (3–5)	
> 200,000	42	(5.30%)	11	(26.2%)	31	(73.8%)		3 (3–4)	
**Residence**							**0.000**		0.889^c^
Urban	406	(50.8%)	94	(23.2%)	312	(76.8%)		4 (3–4)	
Rural	393	(49.2%)	47	(12.0%)	346	(88.0%)		4 (3–4)	
**Distance from residence to hospital**							**0.001**		0.354^b^
< 1 Km	46	(5.80%)	9	(19.6%)	37	(80.4%)		4 (3–4)	
1–5 Km	211	(26.4%)	50	(23.7%)	161	(76.3%)		4 (3–4)	
5–10 Km	129	(16.1%)	31	(24.0%)	98	(76.0%)		3 (3–4)	
> 10 Km	413	(51.7%)	51	(12.3%)	362	(87.7%)		4 (3–4)	
**Time from residence to hospital**							**0.012**		0.497^b^
< 1 h	392	(49.1%)	85	(21.7%)	307	(78.3%)		3 (3–4)	
1–2 h	251	(31.4%)	33	(13.1%)	218	(86.9%)		4 (3–4)	
> 2 h	156	(19.5%)	23	(14.7%)	133	(85.3%)		4 (3–4)	
**Transportation**							**0.014**		0.221^b^
Public transportation	492	(61.6%)	76	(15.4%)	416	(84.6%)		3 (3–4)	
Taxi	133	(16.6%)	35	(26.3%)	98	(73.7%)		4 (3–4)	
Private car	174	(21.8%)	30	(17.2%)	144	(82.8%)		3 (3–4)	
**Displacement status**							**0.000**		0.069^c^
Resident	634	(79.3%)	94	(14.8%)	540	(85.2%)		4 (3–4)	
Internally displaced	165	(20.7%)	47	(28.5%)	118	(71.5%)		3 (3–4)	

*p-value: significant level at 95% confidence interval*,* IQR: Interquartile range*

* Median (IQR)

^*a*^ *Chi-squared test for association between demographic characteristics and recommendation*,* Fisher’s exact test*, ^*b*^ *Kruskal-Wallis test and* ^*c*^ *Mann-whitney U test comparing overall rating across groups*

### Hospitalisation characteristics

Most of the study participants (41.9%) were treated in Kosti Teaching Hospital, and the majority were treated in OB/GYN wards (75.5%). Nearly all the patients stayed in the.

hospital for 1–10 days (98.9%) and had co-patients with them (98.7%). Regarding the admission process, (57.1%) had emergency admission, and only (6.8%) experienced repeated admission within 30 days from the first admission. Most of the patients (64.3%) were admitted for childbirth, and (92.6%) of them underwent a cesarean section. It is important to note that most patients (75%) were not insured.

In terms of the association between the overall rating of quality of care and the hospitalisation characteristics, all the findings were statistically insignificant, except for the hospital, with those admitted in Rabak Hospital having the lowest median score of the overall rating of quality of care; 3 [1–4] with a p-value of < 0.001.

Of the hospitalisation characteristics that were associated with the recommendation of the hospital, only the hospital and ward were statistically significant (p-value < 0.05) (Table 2).

**Table 2:** Hospitalisation characteristics of the study participants admitted to Kosti, ad Douiem, and Rabak teaching Hospital, white nile state, 2023, N: 799

Characteristics	Overall *N*		Recommendation					The overall rating of quality of care	
			No		Yes		*P* value^a^	Median (IQR)	*P* value
**Hospital**							**0.000**		**0.000** ^b^
Kosti Teaching Hospital	335	(41.9%)	73	(21.8%)	262	(78.2%)		4 (3–4)	
Ad Douiem Teaching Hospital	238	(29.8%)	53	(22.3%)	185	(77.7%)		4 (3–5)	
Rabak Teaching Hospital	226	(28.3%)	15	(6.60%)	211	(93.4%)		3 (1–4)	
**Ward**							**0.048**		0.664^b^
Medicine	94	(11.8%)	22	(23.4%)	72	(76.6%)		4 (3–4)	
Surgery	102	(12.8%)	24	(23.5%)	78	(76.5%)		4 (3–4)	
Obstetrics and Gynaecology	603	(75.5%)	95	(15.8%)	508	(84.2%)		3 (3–4)	
**Days of hospital stay**	2 (1–3) *						0.051		0.541^b^
1–10	790	(98.9%)	137	(17.3%)	653	(82.7%)		4 (3–4)	
11–20	7	(0.90%)	4	(57.1%)	3	(42.9%)		3 (2–4)	
21–50	2	(0.30%)	0	(0.00%)	2	(100%)		3 (3–3)	
**Admission Type**							0.145		0.963^c^
New	745	(93.2%)	133	(17.9%)	612	(82.1%)		4 (3–4)	
Repeated	54	(6.80%)	8	(14.8%)	46	(85.2%)		3 (3–4)	
**Admission status**							0.323		0.342^c^
Planned	343	(42.9%)	57	(16.6%)	286	(83.4%)		3 (3–4)	
Emergency	456	(57.1%)	84	(18.4%)	372	(81.6%)		4 (3–4)	
**Reason for admission**							0.529		0.058^b^
New acute disease	170	(21.3%)	29	(17.1%)	141	(82.9%)		4 (3–4)	
Post-operative follow-up	40	(5.00%)	5	(12.5%)	35	(87.5%)		3 (3–4)	
Acute on chronic disease	37	(4.60%)	8	(21.6%)	29	(78.4%)		4 (3–4)	
Trauma/Accident	38	(4.80%)	10	(26.3%)	28	(73.7%)		4 (3–4)	
childbirth	514	(64.3%)	89	(17.3%)	425	(82.7%)		3 (3–4)	
**Health insurance status**							0.492		0.957^c^
Insured	200	(25.0%)	39	(19.5%)	161	(80.5%)		4 (3–4)	
Not insured	599	(75.0%)	102	(17.0%)	497	(83.0%)		4 (3–4)	

*p-value: significant level at 95% confidence interval*,* IQR: Interquartile range*

* Median (IQR)

^*a*^ *Chi-squared test for association between demographic characteristics and recommendation*,* Fisher’s exact test*, ^*b*^ *Kruskal-Wallis test and* ^*c*^ *Mann-Whitney U test comparing overall rating across groups*

### I-PACH survey questions

Regarding the experience of patients with communication with nurses and doctors, more than half of the patients answered “always” in each item. But (30.3%) reported that they could never distinguish between doctors, house officers, and nurses. In terms of the physical environment, most patients reported that the ward where they slept was always (39%) or usually (30.8%) kept clean, and (42.1%) stated that the surrounding area was always kept quiet. Regarding personal privacy, more than half (50.9%) of the participants stated that they never had enough privacy. Most of the patients who experienced pain reported that their pain was always well controlled (63.1%), and the staff always did what they could to help them with their pain (55.5%). Of the patients who received new medications, (51.7%) reported they were always told what the medication was for, and (41.1%) reported that they were never told about the possible side effects in a way that they could understand (Appendix Table 2).

Most (55.1%) of patients reported that they were not given information about symptoms or problems to look out for after leaving the hospital, and (37.2%) of patients reported it was difficult to find their way around the hospital. Almost half (50.3%) of the patients reported it was their first time to be treated at this hospital, and (82.4%) of the participants would recommend the hospital to their families and friends. Most patients (73.3%) had to pay for the hospital stay, and (63.1%) considered it very expensive. Only (2.5%) of the participants rated their overall health as poor and (24.4%) as excellent (Appendix Table 3).

**Fig. 1:**
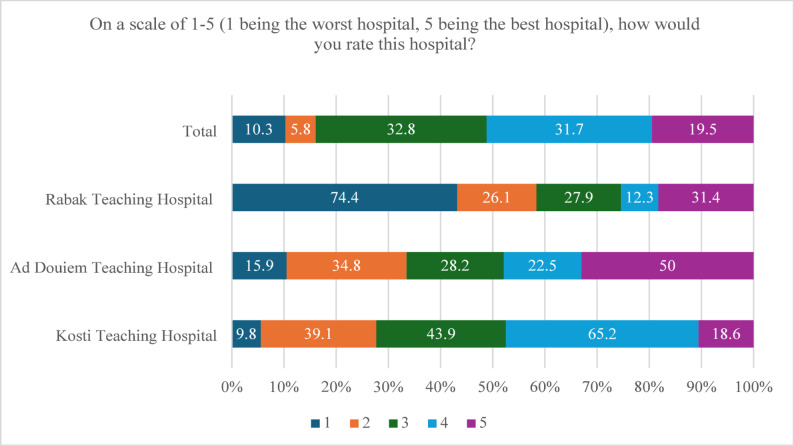
Overall rating of quality of care as reported by the study participants admitted to Kosti, Ad Douiem, and Rabak Teaching Hospital, White Nile state, 2023, N: 799

Regarding the overall rating of quality of care, (31.7%) rated the hospital with “4” out of “5”, and (19.5%) of patients rated the hospital with the best “5”. (Fig. 1).

Regarding items of the I-PACH survey; first time to be treated in the hospital, payment for the hospital stay, considering the hospital too expensive and overall health rating were all statistically significant with the overall rating of quality of care (p-value < 0.05). See (Appendix Table 3) for more details.

### Patients’ experience factors

Three of the five patient experience factors from the I-PACH survey were positively correlated with the overall rating of quality of care, meaning that with every unit increase in the score of one of these factors, the overall ratings of quality of care increase. A weak positive correlation of 0.111 was found between the overall rating with communication with nurses, with a significant p-value of 0.002, and with communication with doctors *r* = 0.073, p-value of 0.040. Physical environment was also positively correlated with the overall rating of quality of care *r* = 0.177, p-value < 0.001. Regarding pain management *r* = 0.021, and medication communication *r* = 0.055 both were not significantly correlated with the overall quality of care (p-value > 0.05) (Appendix Table 4).

### Predictors of the overall rating of quality of care

The model showed that five of the independent variables made a significant contribution to it. The respective hospital was a strong predictor, with patients treated in Rabak Teaching Hospital being more likely to give lower ratings of quality of care (OR 0.227; 95% CI 0.141–0.366; *p* < 0.001). Of the patients’ experience factors, the physical environment was a significant predictor (OR 1.337; 95% CI 1.208–1.480; *p* < 0.001), with each unit increase in scores of physical environments associated with a 24.5% increase in the odds of the higher rating of quality of care. Excellent/Good rating of overall health was also associated with higher odds of a higher rating of quality of care (OR 2.303; 95% CI 1.458–3.638; *p* 0.000) (Table 3).

**Table 3:** Binomial regression model examining the predictors of overall rating of quality of care across the study participants admitted to Kosti ad Douiem, and Rabak teaching Hospital, white nile state, 2023, N: 799

Variable	B	S.E.	*P* value	Odd ratio	95% C.I.for odd ratio	
					Lower	Upper
**Hospital name** (ref: Kosti teaching hospital)			**0.000**			
Ad Douiem teaching hospital	−0.638	0.245	**0.009**	0.528	0.327	0.853
Rabak Teaching Hospital	−1.482	0.243	**0.000**	0.227	0.141	0.366
**First time to be treated in this hospital?** (ref: No)	−0.467	0.182	**0.010**	0.627	0.438	0.896
**Do you consider this hospital stay too expensive?** (ref: No)	−0.595	0.194	**0.002**	0.551	0.377	0.807
**How would you rate your overall health?** (ref: poor/fair)	0.834	0.233	**0.000**	2.303	1.458	3.638
**Communication with nurses**	0.066	0.047	0.161	1.068	0.974	1.170
**Communication with doctors**	0.005	0.058	0.936	1.005	0.896	1.126
**Physical environment**	0.219	0.060	**0.000**	1.245	1.108	1.399

*Classification percentage correct*,* 65.2%; −2 log likelihood*,* 730.446*^*a*^; *Cox & Snell R Square*,* 0.130; Nagelkerke R Square*,* 0.174; Hosmer and Lemeshow*,* 0.768*

*B: effect estimate; S.E: Standard error; C.I: Confidence interval; p-value: significant level at 95% confidence interval*

### Predictors of recommendation of the hospitals

The model shows that the respective hospital was a strong predictor for the recommendation; the odds for recommending the hospital was 3.234 greater among patients treated in Rabak teaching than those treated in Kosti Teaching Hospitals (95% CI 1.710–6.118; *p* < 0.001). Regarding education, patients with secondary and higher education had lower odds of recommending the hospital than those who received lower than primary education. There was also a significant tendency for displaced patients (OR 0.561; 95% CI 0.358–0.881; *p* 0.012) to not recommend the hospital. Using medicine as a reference, patients admitted to the OB/GYN ward had significantly higher odds of recommending the hospital (OR 2.209; 95% CI 1.032–4.728; p 0.041). Patients in the surgery wards also had higher odds (OR 1.153; 95% CI 0.573–2.318; p 0.690), but the result was not statistically significant (Table 4). See (Appendix Table 5) for the complete regression table.

**Table 4:** Binomial regression model examining the predictors of recommending the hospital across the study participants admitted to Kosti, ad Douiem, and Rabak teaching Hospital, white nile state, 2023, N: 799

Variable	B	S.E.	*P* value	Odd ratio	95% C.I.for odd ratio	
					Lower	Upper
**Gender** (ref: Male)	−0.112	0.372	0.764	0.894	0.431	1.854
**Education** (ref: Lower than primary)			**0.025**			
Primary School	−0.216	0.302	0.474	0.806	0.446	1.456
Secondary School	−0.648	0.306	**0.034**	0.523	0.287	0.953
University and above	−0.963	0.366	**0.009**	0.382	0.186	0.783
**Displacement** (ref: resident)	−0.578	0.230	**0.012**	0.561	0.358	0.881
**Hospital** (ref: Kosti Teaching Hospital)			**0.000**			
Ad Douiem Teaching Hospital	−0.122	0.241	0.614	0.886	0.552	1.421
Rabak Teaching Hospital	1.174	0.325	**0.000**	3.234	1.710	6.118
**Ward** (ref: Medicine)			0.102			
Surgery	0.142	0.356	0.690	1.153	0.573	2.318
Obstetrics and Gynaecology	0.793	0.388	**0.041**	2.209	1.032	4.728

*Classification percentage correct*,* 82%; −2 log likelihood*,* 678.780*^*a*^; *Cox & Snell R Square*,* 0.079; Nagelkerke R Square*,* 0.131; Hosmer and Lemeshow*,* 0.332*

*B: effect estimate; S.E: Standard error; C.I: Confidence interval; p-value: significant level at 95% confidence interval*

## Discussion

This study aimed to evaluate patients’ experience with healthcare quality and the predictors of quality of care from their perspective. It highlighted specific areas for quality improvement that are necessary for a good patient experience. The study found that the overall rating of quality of care was low compared to other studies; however, most of the patients would recommend the hospital to their families and friends.

The findings indicate that the overall ratings of the quality of inpatient care were generally low, as only 19.5% rated the quality of care as the best and 10.3% rated the quality as the worst; this is in contrast with another study conducted in rural China using the same scale, where 30.7% of the participants rated the quality as the best and only 0.9% rated the quality as the worst [22]. Another study examined the quality of care in outpatient services in Chinese public hospitals also reported high-quality ratings among patients [23]. This huge difference may be attributed to the fact that China is a high-income country and has better healthcare quality.

However, a study conducted in LMICs found that only a fifth of their participants reported excellent quality of care [24]. Other studies conducted in Ghana and Ethiopia also reported low levels of quality [25–27]. While Sudan is a low-income country and is experiencing armed conflict, our findings align with a systematic review of the quality of health systems in humanitarian settings, suggesting lower levels of satisfaction with healthcare quality in humanitarian settings [10].

This low rating of quality of care can be explained by the fact that most of our participants belonged to vulnerable groups with incomes falling below the poverty line, and vulnerable groups are known to receive the poorest quality of care [2]., or because of the already fragile health system in Sudan, and the insufficient quality of care received [28].

However, suppose we tried to explain the variety in the quality of care ratings in our results alone. In that case, we can notice that half of the patients rated the quality as “4 or 5 out of 5”, and the other half rated the quality between “1–3 out of 5”. So, half of the patients assigned low rating scores, and the other half assigned high rating scores, despite reports suggesting low quality of care in Sudan [28].

This can be explained by the low expectations cycle, in which the patient adapts to poor quality of care, then develops low expectations towards the health services, hence a higher rate of satisfaction with the poor quality of care, leading to demands of lesser quality, and as a result, less pressure is applied to the health system managers to deliver high quality of care, thus decreasing the opportunities for improvement [29]. Patients from low-income countries generally have lower expectations regarding quality of care [29], particularly in contexts such as Sudan, where the ongoing armed conflict has severely disrupted the healthcare system [30]. Understanding patients’ expectations and working towards raising them is crucial, as they represent a key determinant in improving the quality of care. Higher expectations are often associated with a lower perception of care, thereby creating an opportunity for constructive feedback [31]. Some scholars argue that aligning patients’ expectations with the available resources by lowering their expectations may prevent dissatisfaction with healthcare services [32]. However, this argument has an equivocal impact: It may be valid in settings where the technical quality of care provided is already optimal and resource limitations prevent further improvements. Conversely, it risks hindering progress in other aspects of care, such as communication and counselling. Moreover, while this argument may work in high-income countries, where the overall quality of care is comparatively high, it is less applicable in low-income contexts, where significant gaps in quality remain [2].

Self-perceived ratings of health were a significant predictor of quality of care. Patients who rated their health as excellent or good tended to give higher ratings for quality of care. A possible explanation is that people with good health are more optimistic, and they require fewer services from the health system. On the other hand, people with bad health conditions cannot tolerate poor quality, because they need more care, and they put their trust in the health system, in the hope of improving their health status [29]. However, the relationship between health outcomes and quality of care is bidirectional; it is possible that patients with good health received a high quality of care.

Physical environment was also found to be a strong predictor of the overall rating of quality of care. Many studies also found that the physical environment was a significant predictor of quality of care [22, 23, 25]. Thus, it can be considered an important area for quality improvement. Public hospitals are known to have a physical environment of low quality; in our study, a considerable proportion of the patients reported bad experiences with the hospital’s cleanliness and quietness. This is consistent with findings from other studies conducted in Ethiopia [25, 27].

Another important finding is that patients who were treated in the hospital for the first time were more likely to give lower ratings for the hospital. This may be explained by the fact that those people come with higher expectations than the patients who were accustomed to the hospital services, and thus, they will not tolerate the poor quality.

It’s important to note that none of the patients’ demographics were found to influence the overall ratings of quality of care. This is explained by a previous study that highlighted that the care-related characteristics were more important in predicting the overall rating of care than the sociodemographic characteristics [23]. However, other studies have controversially suggested the importance of these characteristics in influencing the overall quality [25–27].

Despite the low ratings of quality of care, 82.4% would recommend the hospital. The odds of recommending the hospital were higher among people who were less educated. This can be explained by the fact that less educated patients lack the perception of high-quality care. Those patients are more likely to come from lower socioeconomic backgrounds, and they may have repeated exposure to low-quality services, thus having low expectations, and therefore they tend to find the poor quality of care satisfactory [2, 29].

Although displacement was not significantly linked to the overall ratings of quality of care, internally displaced persons (IDPs) were less likely to recommend the hospital. Most of the IDPs in our study were displaced from Khartoum state, where the quality of health services was better, with more specialised hospitals, and more trained healthcare professionals available [12]. So, they were less likely to be satisfied with the health services in the hospitals of White Nile State, even if the services were satisfactory for the residents. It is important to note that Sudan’s health system has undergone decentralisation, but it is still experiencing challenges in the implementation, resulting in disproportionate distribution, with nearly 80% of the healthcare services concentrated in Khartoum state, and neglecting the other states [11, 33].

Interestingly, patients admitted to Rabak Teaching Hospital tended to give lower ratings for quality of care; however, their odds of recommending the hospital were three times higher than those of their counterparts in Kosti Teaching Hospital. This may be because these patients have no other choice but to be treated in this hospital, considering the difficulties in accessing better health services.

Ward of admission was not identified as a predictor of the overall quality of care; however, it was found to be a significant predictor of recommending the hospital. Interestingly, patients admitted to the OB/GYN ward were twice as likely to recommend the hospital compared to patients admitted to the medicine ward. This may be explained by the fact that childbirth is often perceived as a positive experience by women [34]. Patel et al. concluded that obstetricians and gynaecologists received higher satisfaction ratings, which were associated with friendly and caring attitudes [35]. In contrast, other studies have found that patients’ ratings of quality of care in OB/GYN wards were lower than those in medical or surgical wards, highlighting the special considerations that should be taken into account when addressing the experiences of obstetric patients [36, 37].

Regarding patients’ experience with specific factors, the results showed high ratings for communication with nurses and doctors, and with each unit increase in scores of communication with nurses and doctors, there was an increase in the overall rating of quality of care. However, the regression model excludes both from the predictors of the overall rating of quality of care, possibly overshadowed by other strong predictors, i.e. physical environment. This does not disprove the importance of communication with the staff. Many studies found that communication with nurses was a strong predictor of quality of care, as well as communication with doctors [22, 38].

The core of patient-centred care is to inform and involve patients in their care decisions. However, our study found that nearly half of the patients who received new medications were not informed about their side effects in a way that was understandable to them. Also, more than half of the study participants did not receive proper counselling about their health condition after leaving the hospital. This finding is consistent with another study examining patient experience, as patients reported that they were not well informed about their health conditions [27].

A considerable number of patients could not distinguish doctors from nurses; this may be due to the absence of clear dress code guidelines for the health staff. The importance of formal clothes is that it increases patients’ confidence in the treating physician [39]. Another important point to highlight is that more than a third of the study participants reported that it was hard to find their way around the hospital. This may be due to the absence of hospital indoor maps or to the improper labelling of the hospital parts.

In contrast to a study conducted in Ethiopia, where the satisfaction with privacy was higher among the patients [25], our study reported that more than half of the patients stated they had not experienced enough privacy. This may be due to the absence of splits and curtains between beds in the wards of those hospitals.

Access to healthcare is an important aspect to be considered, even before the eruption of the armed conflict; access to healthcare was challenging for Sudanese patients. A report by UNICEF explained that 80% of the Sudanese population accesses health services within one hour of travelling, and when they access the care, it is of low quality [28]. This percentage supports our findings, as half of the study participants travelled one hour or more to reach the hospital. Furthermore, half of the participants travelled more than 10 Km to reach the hospital. Which is two times more than the recommended travel distance by the WHO, which is 5 Km [40].

It is worth noting that nearly two-thirds of the patients had to pay for the hospital and most of them found it expensive. In White Nile state, the cost of surgical procedures far exceeds the income level of the patients [41]. While most of our patients had a low-income level, out-of-pocket payments would be hard for them in the absence of health insurance. Even the insured patients would suffer from health-related expenses because the national health insurance program was frozen due to the ongoing armed conflict [42]. The results of our study show that patients who found the hospital stay expensive gave lower ratings of quality of care, and this emphasises the importance of ensuring that health care is available for all, regardless of socioeconomic conditions, to achieve a high-quality health system [2].

Another important finding is that 69.1% of the IDPs live in the host communities, while humanitarian aid focuses primarily on the displacement and refugee camps [43]. Reports from White Nile state hospitals showed that most of the IDPs are facing difficulties in accessing and affording surgical and obstetrical care. Some of these hospitals launched an initiative of free caesarean and normal delivery services for the IDPs. However, these services are threatened to cease due to the financial constraints [41].

## Conclusion

To our knowledge, this is the first study assessing patients’ experience with quality of care in Sudan, and during the armed conflict. The study has explored patients’ experiences in various aspects of care as well as the overall rating of quality of healthcare. The overall rating of quality of care was found to be relatively low compared with other studies. The study revealed that the physical environment strongly predicted quality of care. Thus, attention should be focused on improving the quality of the physical environment. The study also highlights problems in counselling, privacy, and access to healthcare. These findings can help policymakers design health systems more aligned with patients’ preferences.

### Limitations

Although our study has several findings that could be implemented to advance the quality of care not only in White Nile State but in Sudan as a whole, it has several limitations that should be addressed. One is the quantitative nature of the study. It would have been more appropriate to use a mixed-method approach to better explore patients’ experiences and identify themes that were not captured by the quantitative design.

The study area was limited to White Nile State, which restricted the generalizability of the results. A nationwide study would have been more appropriate. However, since all health facilities in Sudan face similar challenges, the findings of this study could significantly contribute to policy drafting and strategic planning to enhance the quality of care in other states.

Furthermore, the sampling technique was non-probability consecutive sampling, and this is a significant limitation as the use of random sampling was hindered due to the unavailability of a structured patient database, ID numbers and numbered beds. Thus, not all the study population had an equal chance of participating; this can introduce significant selection bias. It might over-represent patients admitted during the data collection period, also limiting the generalisability of the findings. Future studies can use cluster sampling techniques to reduce the selection bias. The study aimed to assess patients’ experiences with inpatient care, specifically in the wards. Future studies should investigate patients’ experiences with outpatient care, critical care, radiological and laboratory services and other aspects of care provided within the hospitals.

Lastly, although we performed forward and backwards translation and a pilot study for the I-PAHC questionnaire, a psychometric test for the internal consistency and validity of the I-PAHC in the Sudanese Arabic context was not performed. This linguistic difference may have influenced how patients understood different items and thus affected the precision of our estimates. While our pilot confirmed basic clarity, future studies should validate the instrument to strengthen its use.

## Supplementary Information

Supplementary Material 1.

## Data Availability

All data relevant to the study are de-identified and included in the article or uploaded as supplementary information.

## Abbreviations

FMOH: Federal Ministry of Health
I-PACH: In-Patient Assessment of Health Care survey
LMICs: Low- and middle-income countries
UNICEF: United Nations International Children’s Emergency Fund
WHO: World Health Organisation
IDPs: Internally Displaced Persons
SDG: Sudanese Pound
OB/GYN: Obstetrics and Gynaecology

## Declarations

### Provenance and peer review

Not commissioned; externally peer-reviewed.
