# Supplementary material for: Their voices matter: assessing patients’ experience with healthcare quality in a war-torn country
Source: Confl Health. 2025 Dec 8;19:95. doi: 10.1186/s13031-025-00739-0 (PMC12729083; doi:10.1186/s13031-025-00739-0)
Supplement: Supplementary file 1 — Supplementary Material 1. [file 13031_2025_739_MOESM1_ESM.docx]

Appendix Table 1: Characteristics of Internally Displaced Persons Admitted to Kosti, Ad Douiem, and Rabak Teaching Hospital, White Nile State, 2023, N:165

| **Variable** | **N** | **%** |
| --- | --- | --- |
| **Where did you displace from?** |  |  |
| Khartoum | 150 | (90.9%) |
| North Kordufan | 4 | (2.40%) |
| Middle Darfur | 2 | (1.20%) |
| North Darfur | 2 | (1.20%) |
| East Darfur | 2 | (1.20%) |
| West Darfur | 2 | (1.20%) |
| South Darfur | 3 | (1.80%) |
| **Where are you currently staying?** |  |  |
| In a refugee camp | 26 | (15.8%) |
| Host community | 114 | (69.1%) |
| Rent house | 24 | (14.5%) |
| Owned | 1 | (0.60%) |

Appendix Table 2: I-PACH survey questions (scale questions), as reported by the study participants admitted to Kosti, Ad Douiem, and Rabak Teaching Hospital, White Nile state, 2023, N: 799

| **Question** | Always | | Usually | | Sometimes | | Never | | Total |
| --- | --- | --- | --- | --- | --- | --- | --- | --- | --- |
|  | N % | | | | | | | |  |
| **Communication with nurses (1-3)** | | | | | | | | | |
| 1. During this hospital stay, how often did **nurses** treat you with courtesy and respect? | 477 | (59.7%) | 179 | (22.4%) | 99 | (12.4%) | 44 | (5.50%) | 799 |
| 2. During this hospital stay, how often did nurses listen carefully to you? | 510 | (63.8%) | 143 | (17.9%) | 88 | (11.0%) | 58 | (7.30%) |  |
| 3. During this hospital stay, how often did nurses explain your medical condition in a way you could understand? | 416 | (52.1%) | 147 | (18.4%) | 66 | (8.30%) | 170 | (21.3%) |  |
| **Communication with doctors (4-6)** | | | | | | | | | |
| 4. During this hospital stay, how often did doctors treat you with courtesy and respect? | 620 | (77.6%) | 116 | (14.5%) | 43 | (5.40%) | 20 | (2.50%) | 799 |
| 5. During this hospital stay, how often did doctors listen carefully to you? | 598 | (74.8%) | 139 | (17.4%) | 34 | (4.30%) | 28 | (3.50%) |  |
| 6. During this hospital stay, how often did doctors explain your medical condition in a way you could understand? | 517 | (64.7%) | 128 | (16.0%) | 66 | (8.30%) | 88 | (11.0%) |  |
| 7. I could distinguish between doctors/health officers and nurses | 366 | (45.8%) | 97 | (12.1%) | 94 | (11.8%) | 242 | (30.3%) | 799 |
| **Physical environment (8-9)** | | | | | | | | | |
| 8. During this hospital stay how often was the room you were sleeping in kept clean? | 312 | (39.0%) | 246 | (30.8%) | 152 | (19.0%) | 89 | (11.1%) | 799 |
| 9. During this hospital stay how often was the area around you quite at night? | 336 | (42.1%) | 243 | (30.4%) | 113 | (14.1%) | 107 | (13.4%) |  |
| 10. During this hospital stay how often did the staff make sure you have enough personal privacy? | 80 | (10.0%) | 203 | (25.4%) | 109 | (13.6%) | 407 | (50.9%) |  |
| **Pain management (12-13)** | | | | | | | | | |
| 11. During this hospital stay, did you experience any pain? | Yes | 339 | (42.4%) |  |  |  |  |  | 799 |
|  | No | 460 | (57.6%) |  |  |  |  |  |  |
| 12. During this hospital stay, how often was your pain well controlled? | 214 | (63.1%) | 70 | (20.6%) | 29 | (8.60%) | 26 | (7.70%) | 339 |
| 13. During this hospital stay, how often did the staff do everything they could help you with your pain? | 188 | (55.5%) | 79 | (23.3%) | 27 | (8.00%) | 45 | (13.3%) |  |
| **Medication communication (15-16)** | | | | | | | | | |
| 14. During this hospital stay, were you given any medication that you had not taken before? | Yes | 292 | (36.5%) |  |  |  |  |  | 799 |
|  | No | 507 | (63.5%) |  |  |  |  |  |  |
| 15. Before giving you any new medication, how often did staff tell you what the medicine was for? | 151 | (51.7%) | 79 | (27.1%) | 28 | (9.60%) | 34 | (11.6%) | 292 |
| 16. Before giving you any new medication, how often did staff describe possible side effects in a way you could understand? | 69 | (23.6%) | 68 | (23.3%) | 35 | (12.0%) | 120 | (41.1%) |  |

Appendix Table 3: I-PACH survey questions (non-scale items) as reported by the study participants admitted to Kosti, Ad Douiem, and Rabak Teaching Hospital, White Nile state, 2023, N: 799

| **Question** | **Overall N** | | **The overall rating of the quality of care** | |
| --- | --- | --- | --- | --- |
|  |  |  | **Median (IQR)** | **P value** |
| **Were you given information in a way you could understand what symptoms or health problems to look out after you leave the hospital?** |  |  |  | 0.096 |
| Yes | 359 | (44.9%) | 4 (3-4) |  |
| No | 440 | (55.1%) | 3 (3-4) |  |
| **Was it easy to find your way around the hospital?** |  |  |  | 0.700 |
| Yes | 502 | (62.8%) | 4 (3-4) |  |
| No | 297 | (37.2%) | 4 (3-4) |  |
| **Is this your first time being treated at this hospital?** |  |  |  | **0.009** |
| Yes | 402 | (50.3%) | 3 (3-4) |  |
| No | 397 | (49.7%) | 4 (3-4) |  |
| **Would you recommend this hospital to your friends and**  **family?** |  |  |  | **0.000** |
| Yes | 658 | (82.4%) | 4 (3-4) |  |
| No | 141 | (17.6%) | 3 (2-4) |  |
| **Did you have to pay for this hospital stay?** |  |  |  | **0.044** |
| Yes | 586 | (73.3%) | 4 (3-4) |  |
| No | 213 | (26.7%) | 4 (3-5) |  |
| **Do you consider this hospital stay too expensive?** |  |  |  | **0.000** |
| Yes | 370 | (63.1%) | 3 (3-4) |  |
| No | 216 | (36.9%) | 4 (3-5) |  |
| **How would you rate your overall health? (Self-perceived health)** |  |  |  | **0.000** |
| Poor | 20 | (2.50%) | 3 (1-3) |  |
| Fair | 128 | (16.0%) | 3 (3-4) |  |
| Good | 456 | (57.1%) | 3 (3-4) |  |
| Excellent | 195 | (24.4%) | 4 (3-5) |  |
| *p-value: significant level at 95% confidence interval, IQR: Interquartile range* | | | | |

Appendix Table 4: Measure of correlation between patients' experience factors and overall rating of quality of care using spearman’s-rho test across the study participants admitted to Kosti, Ad Douiem, and Rabak Teaching Hospital, White Nile state, 2023, N: 799

| How would you rate this hospital? (Overall rating of quality of care) | | | |
| --- | --- | --- | --- |
| **Patients’ experience Factors** | **Correlation coefficient (rho)** | **P value** | **N** |
| Communication with nurses | 0.111 | **0.002** | 799 |
| Communication with doctors | 0.073 | **0.040** | 799 |
| Physical environment | 0.117 | **0.000** | 799 |
| Pain management | 0.021 | 0.703 | 339 |
| Medication communication | 0.055 | 0.353 | 292 |

Appendix Table 5: Binomial regression model examining the predictors of recommending the hospital across the study participants admitted to Kosti, Ad Douiem, and Rabak Teaching Hospital, White Nile state, 2023, N: 799

| **Variable** | **B** | **S.E.** | **P value** | **Odd ratio** | **95% C.I.for odd ratio** | |
| --- | --- | --- | --- | --- | --- | --- |
|  |  |  |  |  | **Lower** | **Upper** |
| **Gender** (ref: Male) | -0.112 | 0.372 | 0.764 | 0.894 | 0.431 | 1.854 |
| **Education** (ref: Lower than primary) |  |  | **0.025** |  |  |  |
| Primary School | -0.216 | 0.302 | 0.474 | 0.806 | 0.446 | 1.456 |
| Secondary School | -0.648 | 0.306 | **0.034** | 0.523 | 0.287 | 0.953 |
| University and above | -0.963 | 0.366 | **0.009** | 0.382 | 0.186 | 0.783 |
| **Residence** (ref: urban) | 0.033 | 0.300 | 0.914 | 1.033 | 0.574 | 1.858 |
| **Time from residence to hospital** (ref: <1 h) |  |  | 0.421 |  |  |  |
| 1-2 h | 0.349 | 0.274 | 0.202 | 1.417 | 0.829 | 2.423 |
| >2 h | 0.119 | 0.354 | 0.737 | 1.126 | 0.562 | 2.256 |
| **Distance** (ref: <1 Km) |  |  | 0.838 |  |  |  |
| 1-5 Km | -0.187 | 0.429 | 0.662 | 0.829 | 0.358 | 1.921 |
| 5-10 Km | -0.315 | 0.457 | 0.491 | 0.730 | 0.298 | 1.788 |
| >10 Km | -0.071 | 0.503 | 0.888 | 0.932 | 0.348 | 2.495 |
| **Transportation** (ref: Public transportation) |  |  | 0.795 |  |  |  |
| Taxi | -0.181 | 0.269 | 0.501 | 0.835 | 0.493 | 1.414 |
| Private car | -0.068 | 0.254 | 0.790 | 0.935 | 0.568 | 1.538 |
| **Displacement** (ref: resident) | -0.578 | 0.230 | **0.012** | 0.561 | 0.358 | 0.881 |
| **Hospital** (ref: Kosti Teaching Hospital) |  |  | **0.000** |  |  |  |
| Ad Douiem Teaching Hospital | -0.122 | 0.241 | 0.614 | 0.886 | 0.552 | 1.421 |
| Rabak Teaching Hospital | 1.174 | 0.325 | **0.000** | 3.234 | 1.710 | 6.118 |
| **Ward** (ref: Medicine) |  |  | 0.102 |  |  |  |
| Surgery | 0.142 | 0.356 | 0.690 | 1.153 | 0.573 | 2.318 |
| Obstetrics and Gynaecology | 0.793 | 0.388 | **0.041** | 2.209 | 1.032 | 4.728 |
| Constant | 1.485 | 0.542 | 0.006 | 4.415 |  |  |
| *Classification percentage correct, 82%; -2 log likelihood, 678.780^a^; Cox & Snell R Square, 0.079; Nagelkerke R Square, 0.131; Hosmer and Lemeshow, 0.332* | | | | | | |
| *B: effect estimate; S.E: Standard error; C.I: Confidence interval; p-value: significant level at 95% confidence interval* | | | | | | |

# Sample Size Calculations

Appendix Table 6: Kosti Teaching Hospital Admission Rate and Sample Size

|  | **Medicine** | **Surgery** | **Obstetrics and gynaecology** | **Total** | **Sample size** |
| --- | --- | --- | --- | --- | --- |
| **Admission for 6 months** | 1407 | 1187 | 4571 | 7165 |  |
| **Average of 6 months** | 235 | 198 | 762 | **1195** |  |
| **Percentage of Total** | 19.7 | 16.6 | 63.8 | 100% |  |
| **Sample size** | 59 | 50 | 191 |  | **300** |

Appendix Table 7: Rabak Teaching Hospital Admission Rate and Sample Size

|  | **Medicine** | **Surgery** | **Obstetrics and gynaecology** | **Total** | **sample size** |
| --- | --- | --- | --- | --- | --- |
| **Admission for 6 months** | 82 | 317 | 2699 | 3098 |  |
| **Average of 6 months** | 14 | 53 | 450 | **517** |  |
| **Percentage of Total** | 2.7 | 10.3 | 87 | 100% |  |
| **Sample size** | 6 | 23 | 197 |  | **226** |

Appendix Table 8: Ad Douiem Teaching Hospital Admission Rate and Sample Size

|  | **Medicine** | **Surgery** | **Obstetrics and Gynaecology** | **Total** | **sample size** |
| --- | --- | --- | --- | --- | --- |
| **Admission for 6 months** | 204 | 214 | 1878 | 2296 |  |
| **Average of 6 months** | 34 | 36 | 313 | **383** |  |
| **Percentage of Total** | 8.9 | 9.4 | 81.7 | 100% |  |
| **Sample size** | 17 | 18 | 161 |  | **196** |
